# Radiologic Pseudoprogression Associated With a Positive Response in a Patient With Multiple Myeloma Treated With a BCMA×CD3 Bispecific Antibody: A Case Report

**DOI:** 10.1002/jha2.70168

**Published:** 2025-11-06

**Authors:** Abdullah M. Khan, Glenn S. Kroog, Tito Roccia, Kate Knorr, Anita Boyapati, Naresh Bumma

**Affiliations:** ^1^ Division of Hematology Department of Internal Medicine The Ohio State University Comprehensive Cancer Center Columbus Ohio USA; ^2^ Regeneron Pharmaceuticals Inc Tarrytown New York USA

**Keywords:** bispecific T‐cell engager, case report, immunotherapy, multiple myeloma, pseudoprogression

## Abstract

Pseudoprogression is characterised by an increase in tumour size, driven by an influx of inflammatory cells, followed by regression. This phenomenon has rarely been reported in multiple myeloma (MM), despite increased use of immunotherapy approaches that have been associated with pseudoprogression in other malignancies. We report a case of pseudoprogression in a female in her early seventies with relapsed/refractory MM who was treated with the BCMA×CD3 bispecific antibody linvoseltamab. At study Day 28, she was hospitalised with acute bone pain and a new fracture, and a positron emission tomography/computed tomography scan suggested disease progression. However, biochemical markers of disease burden (serum M‐protein, kappa light chain) had decreased from baseline, and therefore linvoseltamab was continued. At Day 77, M‐protein was absent and the patient had achieved complete biochemical response, but she had to discontinue linvoseltamab due to infectious complications. A subsequent scan on Day 86 demonstrated marked improvement in osseous lesions. Despite being off therapy, the disease did not progress for 2 years. Assessment of T‐cell activation and proinflammatory cytokine production indicated increased T‐cell activation concurrent with onset of radiologic pseudoprogression. To our knowledge, this is the first description of radiologic pseudoprogression in MM with anti‐BCMA bispecific antibody therapy.

## 1 Introduction

Multiple myeloma (MM) is a plasma cell malignancy accounting for ∼10% of haematologic cancers [1]. MM presents with elevated calcium, renal dysfunction, anaemia, and/or bone lesions [2]. Radiologic imaging is an important modality for disease assessment, and in 2014, the International Myeloma Working Group (IMWG) updated the imaging criteria to improve evaluation of bone and extramedullary disease using computed tomography (CT), magnetic resonance imaging (MRI), and positron emission tomography (PET)/CT [2]. PET/CT functional and morphologic imaging is now established for the evaluation of extramedullary tumour burden and bone disease as well as providing prognostic information based on the presence/absence of residual focal lesions after therapy.

Treatment for MM continues to advance yet outcomes remain poor for many patients with relapsed/refractory disease. The need to improve outcomes has prompted development of new treatments that redirect the immune system to eliminate malignant cells. Chimeric antigen receptor (CAR) T cells and bispecific antibodies have demonstrated favourable efficacy in MM and are emerging as standard‐of‐care options for many patients in the relapsed/refractory setting [3].

Similar to other solid and haematologic malignancies, the adoption of immunotherapy for MM introduces the possibility of pseudoprogression of extramedullary or bone lesions. This phenomenon is characterised by the increase in size (radiographic signal) of a primary lesion, or emergence of a new lesion, with subsequent tumour regression [4]. The increase in lesion size is attributable to an influx of activated inflammatory cells into the tumour environment, rather than true progression, and results in localised oedema, haemorrhage, and necrosis [4]. As pseudoprogression can confound assessment of treatment effectiveness, potentially leading to discontinuation of effective treatment, it is important that healthcare providers are aware of the possibility of this event.

Pseudoprogression has been described with immunotherapy in solid tumours [4, 5], most commonly with checkpoint inhibitors [4], and has also been reported with some T‐cell‐engaging therapies for haematologic malignancies [6, 7]. Leipold et al. described pseudoprogression in a patient who received anti‐B‐cell maturation antigen (BCMA) CAR T‐cell therapy (idecabtagene vicleucel) and a G protein‐coupled receptor, class C, group 5, member D (GPRC5D) × cluster of differentiation 3 (CD3) bispecific antibody (talquetamab), yet this phenomenon has not been reported with anti‐BCMA bispecific antibodies in MM [8, 9]. Here, we describe a case of pseudoprogression during treatment with linvoseltamab, a BCMA×CD3 bispecific antibody.

## 2 Case Presentation

A female ∼70 years old was diagnosed with International Staging System stage II immunoglobulin A kappa MM and received four prior lines of therapy before enrolment into the LINKER‐MM1 study (NCT03761108; linvoseltamab monotherapy in relapsed/refractory MM): (1) Lenalidomide–dexamethasone, autologous stem cell transplantation (ASCT), and lenalidomide maintenance; (2) bortezomib, lenalidomide, and dexamethasone, a second ASCT, and pomalidomide maintenance; (3) daratumumab, pomalidomide, and dexamethasone; (4) daratumumab, carfilzomib, and dexamethasone.

**FIGURE 1:**
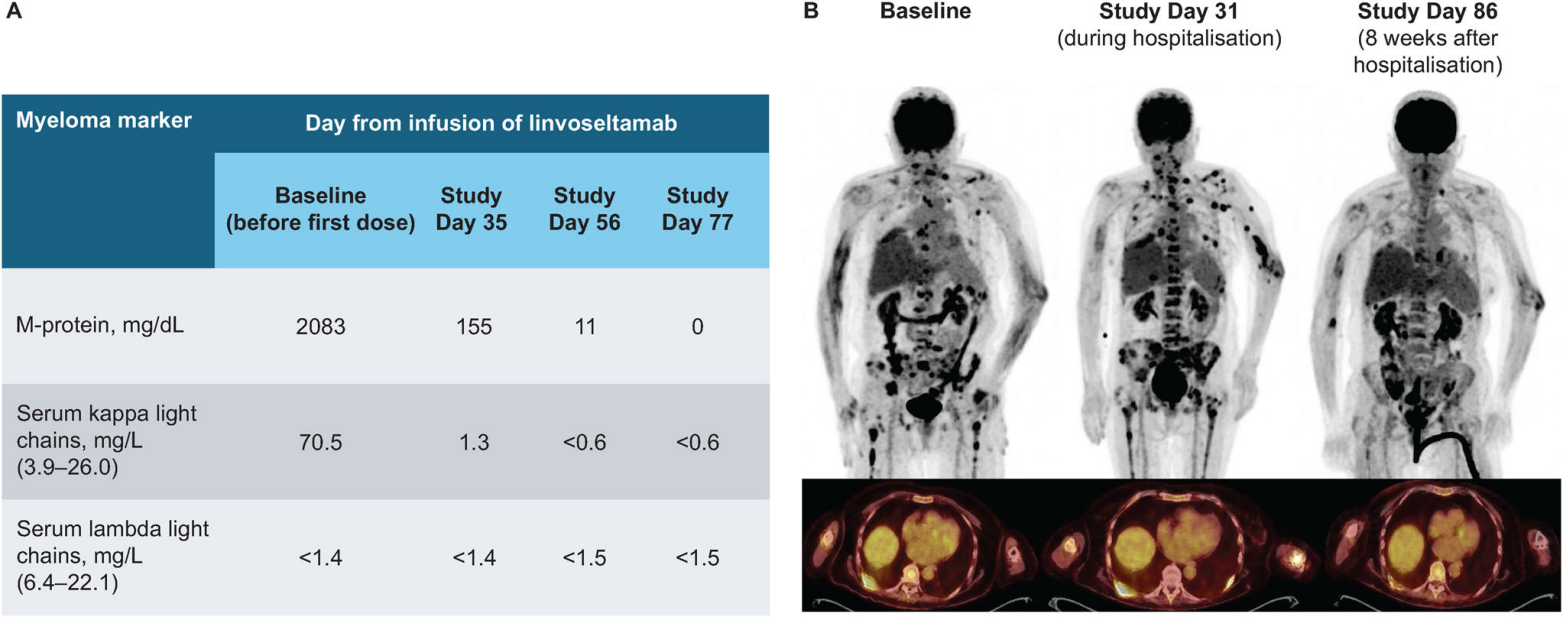
Disease and radiologic assessment of the patient before, during and after linvoseltamab treatment. (A) Myeloma protein markers from baseline (before the first dose of linvoseltamab) to study Day 77. For serum kappa and lambda light chains, the values in brackets represent the normal range, and the value beside “<” represents the lower limit of quantification for that assay. (B) PET/CT scan images prior to initiation of linvoseltamab (left), at the time of hospital admission (centre) and at approximately 8 weeks following hospitalisation (right). New FDG‐avid lesions and increased FDG avidity of existing lesions were observed at hospital admission, but many of these disappeared during the following 8 weeks. A new FDG‐avid lesion was identified in the left humerus at hospital admission, but had resolved at the scan 8 weeks post hospitalisation. FDG, 18F‐fluorodeoxyglucose; PET/CT, positron emission tomography/computed tomography.

Next, in LINKER‐MM1, she received linvoseltamab 5/25 mg step‐up dosing, followed by 200 mg full doses. The patient had high disease burden at baseline (prior to linvoseltamab), based on high concentrations of soluble BCMA (915 ng/mL), elevated serum kappa light chain (70.5 mg/L) (Figure 1A), and PET/CT scan showing multiple bone lesions (Figure 1B, left).

The first linvoseltamab doses were generally well tolerated; no cytokine release syndrome was observed with the initial doses or at any stage during linvoseltamab treatment. One month after starting linvoseltamab (study Day 28), the patient was admitted to the hospital with acute‐onset right groin pain and inability to bear weight. X‐ray of the right hip showed a new non‐displaced pathologic fracture, and multiple lytic lesions previously identified at baseline. She received Augmentin until Day 37 due to concern of a urinary tract infection, and oxycodone (every 6 h as required) for pain management.

Due to the concern for progression, serum/urine MM markers were assessed following hospitalisation (Day 35). M‐protein had decreased from baseline (2083 to 155 mg/dL), as had kappa light chain (70.5 to 1.3 mg/L), indicative of response to treatment (Figure 1A). However, a PET/CT scan was also obtained (Day 31) and suggested disease progression based on both new and worsening osseous lesions throughout the axial and appendicular skeleton (Figure 1B, centre). In addition, an increase in size and 18F‐fluorodeoxyglucose (FDG)‐avidity of existing hypermetabolic left axillary lymph nodes and potential disease at new lymph nodes (left axillary, left posterior shoulder, porta hepatis, and left pelvic mesentery) were observed. Given the discordance between disease markers and PET/CT assessment, the case was discussed with the trial monitor and the patient remained on study treatment.

Two months after starting linvoseltamab (Day 77), the patient discontinued treatment due to an infectious complication; the final linvoseltamab dose was on Day 44. At study discontinuation, the patient was in complete biochemical response. Laboratory tests demonstrated that M‐protein had continued to decrease from Day 35 to 56 and was completely absent by Day 77 (Figure 1A). Similarly, kappa light chain had decreased to <0.6 mg/L by Day 56 and remained at this level at Day 77 (Figure 1A). A PET/CT scan on Day 86 (∼8 weeks after hospitalisation) demonstrated marked improvement of osseous lesions (Figure 1B; right). For example, a lesion along the right posterior eighth rib had a decrease in standardised uptake value to 4.0 (from 5.7) and there was resolution of hypermetabolic lymph nodes above and below the diaphragm, with no new hypermetabolic foci or lymphadenopathy. The patient remained off MM therapy and free from disease progression for 2 years. An MRI taken ∼2 years after the initial hospitalisation event, during the prolonged period of disease regression, also revealed healing of the right‐hip fracture.

**FIGURE 2:**
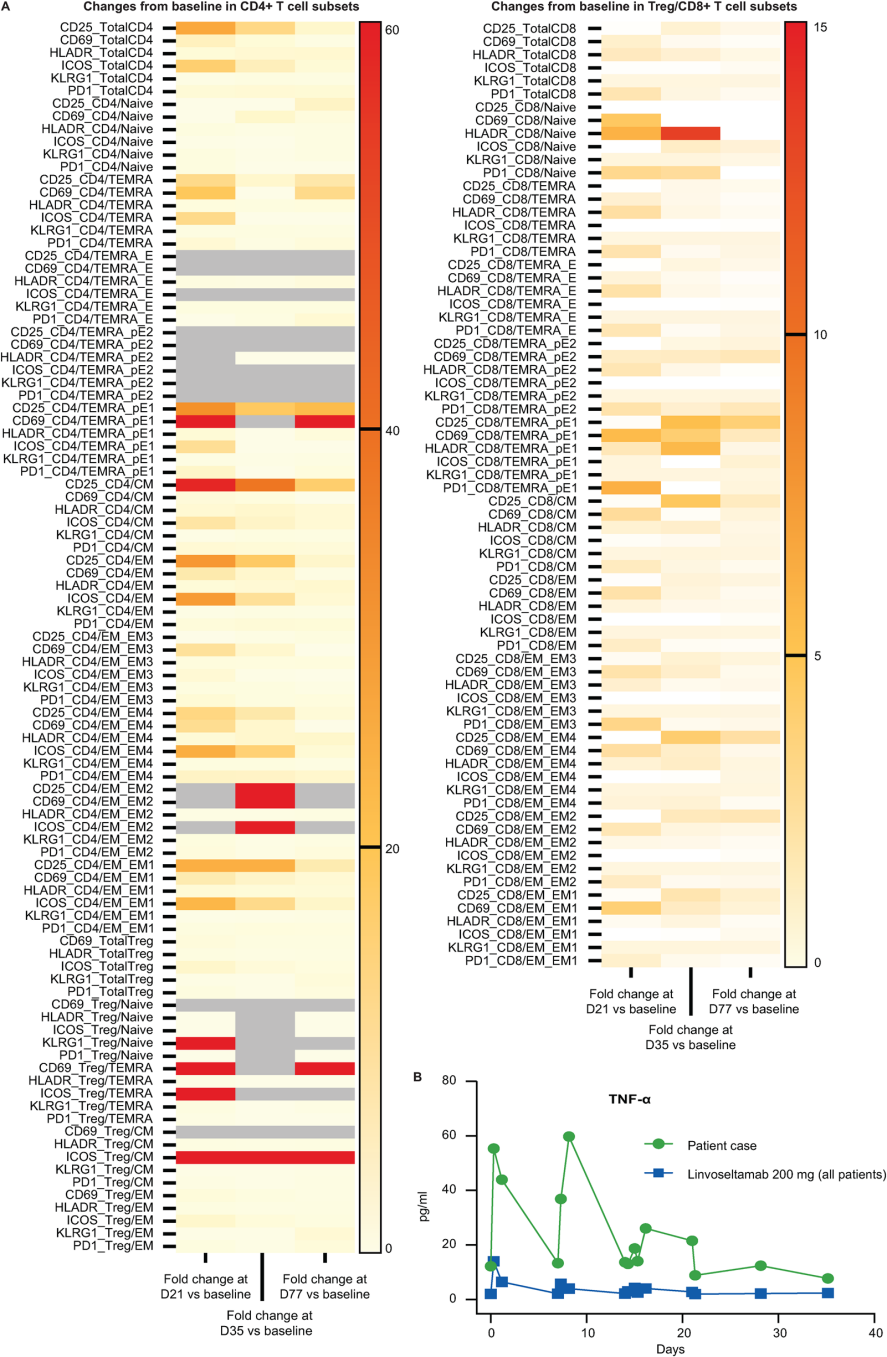
Immunologic findings based on flow cytometry of peripheral blood and measurements of serum cytokine levels. (A) Changes from baseline (before administration of the first dose of linvoseltamab) in CD4^+^ T‐cell subsets and Treg/CD8^+^ T‐cell subsets. (B) Changes in TNF‐α levels to study Day 35; TNF‐α levels in the patient case described are represented by the line with green circles, and the line with blue squares represents the mean TNF‐α levels during this period for all evaluable patients who received linvoseltamab 200 mg. CD, cluster of differentiation; CM, central memory; D, day; EM, effector memory; FU, follow‐up; HLA‐DR, human leukocyte antigen‐DR isotype; ICOS, inducible T‐cell co‐stimulators; KLRG1, killer cell lectin‐like receptor G1; pE, proliferative effector; PD1, programmed cell death 1; TEMRA, terminally differentiated effector memory T cells; TEMRA_E, TEMRA effector; TNF, tumour necrosis factor; Treg, T regulatory; W, week.

Immunologic and cytokine profiles were assessed by peripheral blood flow cytometry and measurement of serum cytokine levels. Before treatment, there was a high proportion of CD8^+^ T cells (72.8%) in peripheral blood relative to CD4^+^ T cells (15.3%). After linvoseltamab initiation, assessments at Days 21 and 35 demonstrated >5‐fold increases from baseline in activated subsets of CD4^+^ and CD8^+^ T cells, with notable rises in CD69^+^ and human leukocyte antigen‐DR^+^ CD8 naïve, CD25^+^ CD4^+^ central memory/effector memory, and CD69^+^ CD4 effector memory cells, at one or both time points (Figure 2A). During the first weeks of treatment, the concentration of tumour necrosis factor (TNF)‐α (cytokine associated with T‐cell activation) was higher in this patient than the mean in the overall cohort of patients receiving 200 mg (Figure 2B). However, TNF‐α concentration decreased over time and was similar to the overall cohort by Day 35.

## 3 Discussion

To our knowledge, this is the first description of radiologic pseudoprogression in a patient with MM following treatment with a BCMA×CD3 bispecific antibody. Pseudoprogression is associated with infiltration of activated immune cells into the tumour microenvironment, which is possible based on the mechanism of action of linvoseltamab [4, 10]. In this case, pseudoprogression coincided with increased levels of activated CD4^+^ T cells, CD8^+^ T cells, and circulating TNF‐α, consistent with T‐cell activation by linvoseltamab [10]. The clinical course supported the occurrence of pseudoprogression, given the subsequent deep and durable response achieved that indicated linvoseltamab was efficacious.

The incidence of pseudoprogression may be related to cancer type, choice of immunotherapy, and criteria used to define pseudoprogression [4, 5]. Relatively high rates have been reported in melanoma and non‐small cell lung cancer (10%–25% and 6%–17%, respectively) [5]. Data in MM are lacking, and guidelines for response assessment in MM do not discuss pseudoprogression [3]. Nevertheless, MM healthcare providers should be aware of pseudoprogression and how to evaluate the possibility of this phenomena in order to facilitate appropriate management decisions [4]. Indeed, our case demonstrates that changing anti‐myeloma therapy was not necessary and the patient benefited from continued linvoseltamab treatment. Similarly, continued talquetamab treatment reduced disease burden in the pseudoprogression case of Leipold et al. [9]. These data support the hypothesis that pseudoprogression may indicate response to immunotherapy [4]. Accurate differentiation between true progression and pseudoprogression is thus critical in preventing discontinuation of beneficial treatment, or continuation of ineffective treatment [5].

Our case demonstrates the potential discordance between radiologic findings and other measures of disease burden in MM. Similar observations were reported by Leipold et al. [9], where a diffusion‐weighted MRI indicated that extramedullary lesions had increased in size during talquetamab treatment, while laboratory assessments demonstrated M‐protein was decreasing. The same case also described pseudoprogression after CAR T‐cell therapy based on FDG and other PET tracer uptake at lung and mediastinal lymph nodes and single‐cell RNA sequencing of bronchoalveolar lavage specimens, in concert with an endobronchial biopsy that showed no evidence of residual MM cells [8, 9]. Collectively, these results highlight the need to consider the full clinical and laboratory picture during the initial weeks of immunotherapy. For patients with MM, protein biomarkers can serve as a guide alongside radiologic findings and other tools to differentiate true progression from pseudoprogression.

In conclusion, here we present the first report of pseudoprogression in a patient receiving treatment with a BCMA×CD3 bispecific antibody. Identification of pseudoprogression, through careful consideration of divergent imaging and biochemical results, enabled extended treatment with linvoseltamab and achievement of deep disease remission for ∼2 years after cessation of therapy. As the anti‐MM armamentarium has expanded to include more T‐cell‐engaging therapies, assessment of the overall clinical picture, rather than relying upon radiologic or laboratory findings in isolation, remains essential for effective patient management.

## Ethics Statement

The protocol for the LINKER‐MM1 study, which this patient participated in, was conducted in accordance with the principles of the Declaration of Helsinki and the International Conference on Harmonization Good Clinical Practice guidelines. The protocol and all amendments were approved by the institutional review board or independent ethics committee of each participating site.

## Consent

The patient provided written consent for participation in the LINKER‐MM1 study.

## Conflicts of Interest

AMK received speaker bureau fees from Amgen and Sanofi, and travel support from BMS. GSK, TR, KK, and AB are employees of, and hold stock in, Regeneron Pharmaceuticals, Inc. NB provided consultancy for Janssen and Sanofi, and received speaker bureau fees from Sanofi.

## Data Availability

Patient personal data will be treated in compliance with all applicable laws and regulations. The sponsor shall take all appropriate measures to safeguard and prevent access to this data by any unauthorised third party. Qualified researchers can request access to study documents that support the methods and findings in this manuscript. Individual anonymised participant data will be considered for sharing (i) once the product and indication have been approved by major health authorities [e.g. Food and Drug Administration (FDA), European Medicines Agency (EMA), Pharmaceuticals and Medical Devices Agency (PMDA), etc.] or development of the product has been discontinued globally for all indications and there are no plans for future development; (ii) if there is legal authority to share the data; and (iii) there is not a reasonable likelihood of participant re‐identification. Requests should be submitted to https://vivli.org/.

## References

[1] D. T. Zhu , A. Park , A. Lai , L. Zhang , H. Attar , and T. R. Rebbeck , “Multiple Myeloma Incidence and Mortality Trends in the United States, 1999–2020,” Scientific Reports 14, no. 1 (2024): 14564. 38914692 10.1038/s41598-024-65590-4 PMC11196710

[2] S. V. Rajkumar , M. A. Dimopoulos , A. Palumbo , et al., “International Myeloma Working Group Updated Criteria for the Diagnosis of Multiple Myeloma,” Lancet Oncology 15, no. 12 (2014): e538–548. 25439696 10.1016/S1470-2045(14)70442-5

[3] Referenced with permission from the NCCN Clinical Practice Guidelines in Oncology (NCCN Guidelines^®^) for multiple myeloma. Version 1.2025. © National Comprehensive Cancer Network, Inc. 2025. All rights reserved. “To View the Most Recent and Complete Version of the Guidelines, Go to NCCN.Org.” NCCN makes no warranties of any kind whatsoever regarding their content, use or application and disclaims any responsibility for their application or use in any way. (1920) Accessed February 19, 2025.

[4] W. Jia , Q. Gao , A. Han , H. Zhu , and J. Yu , “The Potential Mechanism, Recognition and Clinical Significance of Tumor Pseudoprogression After Immunotherapy,” Cancer Biology & Medicine 16, no. 4 (2019): 655–670. 31908886 10.20892/j.issn.2095-3941.2019.0144 PMC6936240

[5] E. S. Waxman and D. L. Gerber , “Pseudoprogression and Immunotherapy Phenomena,” Journal of the Advanced Practitioner in Oncology 11, no. 7 (2020): 723–731. 33575068 10.6004/jadpro.2020.11.7.6 PMC7646636

[6] C. Sortais , S. Cordeil , E. Bourbon , et al., “Flare‐Up Phenomenon or Pseudoprogression After CAR T‐cell Infusion in Non‐Hodgkin Aggressive Lymphomas,” Leukemia & Lymphoma 64, no. 3 (2023): 707–711. 36573418 10.1080/10428194.2022.2161304

[7] W. Bahaj , M. Hegazi , and R. Emmons , “Unveiling Immediate Pseudo‐Progression With Epcoritamab in Lymphoma Treatment: Case Series Exploration,” Transplantation and Cell Therapy 30, no. 2 (2024): S157.

[8] A. M. Leipold , R. A. Werner , J. Düll , et al., “Th17.1 Cell Driven Sarcoidosis‐Like Inflammation After Anti‐BCMA CAR T Cells in Multiple Myeloma,” Leukemia 37, no. 3 (2023): 650–658. 36720972 10.1038/s41375-023-01824-0 PMC9888347

[9] A. Leipold , R. Werner , J. Duell , et al., “Pseudoprogression and Sarcoidosis‐Like Phenomena After CART‐Cells and Bispecific Antibodies in Multiple Myeloma,” Blood 140, no. Suppl 1 (2022): 10020–10021.

[10] D. J. DiLillo , K. Olson , K. Mohrs , et al., “A BCMAxCD3 Bispecific T Cell‐Engaging Antibody Demonstrates Robust Antitumor Efficacy Similar to That of Anti‐BCMA CAR T Cells,” Blood Advances 5, no. 5 (2021): 1291–1304. 33651100 10.1182/bloodadvances.2020002736 PMC7948265

